# Correlation of body composition in early pregnancy on gestational diabetes mellitus under different body weights before pregnancy

**DOI:** 10.3389/fendo.2022.916883

**Published:** 2022-10-28

**Authors:** Li Xintong, Xu Dongmei, Zhang Li, Cao Ruimin, Hao Yide, Cui Lingling, Chen Tingting, Guo Yingying, Li Jiaxin

**Affiliations:** ^1^ Department of Obstetrics and Gynecology, Third Affiliated Hospital of Zhengzhou University, Zhengzhou, China; ^2^ Department of Perinatal Health, Third Affiliated Hospital of Zhengzhou University, Zhengzhou, China; ^3^ Anesthesiology, Xinxiang Medical University, Xinxiang, China; ^4^ Department of Nutrition and Food Hygiene, College of Public Health, Zhengzhou University, Zhengzhou, China

**Keywords:** body mass index, gestational diabetes, bioelectrical impedance assessment, body composition, body fat mass

## Abstract

**Objective:**

The prediction of gestational diabetes mellitus (GDM) by body composition-related indicators in the first trimester was analyzed under different body mass index (BMI) values before pregnancy.

**Methods:**

This was a retrospective analysis of pregnant women who were treated, had documented data, and received regular perinatal care at the Third Affiliated Hospital of Zhengzhou University from January 1, 2021, to December 31, 2021. Women with singleton pregnancies who did not have diabetes before pregnancy were included. In the first trimester (before the 14th week of pregnancy), bioelectric impedance assessment (BIA) was used to analyze body composition-related indicators such as protein levels, mineral levels, fat volume, and the waist-hip fat ratio. The Pearman’s correlation coefficient was used to evaluate the linear relationship between the continuous variables and pre-pregnancy body mass index (BMI). In the univariate body composition analysis, the association with the risk of developing GDM was included in a multivariate analysis using the relative risk and 95% confidence interval obtained from logarithmic binomial regression, and generalized linear regression was used for multivariate regression analysis. Furthermore, the area under the curve (AUC) was calculated by receiver operating characteristic (ROC) curves. The optimal cutoff value of each risk factor was calculated according to the Youden Index.

**Results:**

In a retrospective study consisting of 6698 pregnant women, we collected 1109 cases of gestational diabetes. Total body water (TBW), protein levels, mineral levels, bone mineral content (BMC), body fat mass (BFM), soft lean mass (SLM), fat-free mass (FMM), skeletal muscle mass (SMM), percent body fat (PBF), the waist-hip ratio (WHR), the visceral fat level (VFL), and the basal metabolic rate (BMR) were significantly higher in the GDM group than in the normal group (P<0.05). Under the pre-pregnancy BMI groupings, out of 4157 pregnant women with a BMI <24 kg/m^2^, 456 (10.97%) were diagnosed with GDM, and out of 2541 pregnant women with a BMI ≥24 kg/m^2^, 653 (25.70%) were diagnosed with GDM. In the generalized linear regression model, it was found that in all groups of pregnant women, pre-pregnancy BMI, age, gestational weight gain (GWG) in the first trimester, and weight at the time of the BIA had a certain risk for the onset of GDM. In Model 1, without adjusting for confounders, the body composition indicators were all positively correlated with the risk of GDM. In Model 3, total body water, protein levels, mineral levels, bone mineral content, soft lean mass, fat-free mass, skeletal muscle mass, and the basal metabolic rate were protective factors for GDM. After Model 4 was adjusted for confounders, only the waist-hip ratio was positively associated with GDM onset. Among pregnant women with a pre-pregnancy BMI <24 kg/m^2^, the body composition-related indicators in Model 2 were all related to the onset of GDM. In Model 3, total body water, soft lean mass, fat-free mass, and the basal metabolic rate were negatively correlated with GDM onset. In the body composition analysis of among women with a pre-pregnancy BMI ≥ 24 kg/m^2^, only Model 1 and Model 2 were found to show positive associations with GDM onset. In the prediction model, in the basic data of pregnant women, the area under the receiver operating characteristic curve predicted by gestational weight gain for GDM was the largest (0.795), and its cutoff value was 1.415 kg. In the body composition results, the area under the receiver operating characteristic curve of body fat mass for predicting GDM risk was larger (0.663) in all pregnant women.

**Conclusions:**

Through this retrospective study, it was found that the body composition-related indicators were independently associated with the onset of GDM in both the pre-pregnancy BMI <24 kg/m^2^ and pre-pregnancy BMI ≥24 kg/m^2^ groups. Body fat mass, the visceral fat level, and the waist-hip ratio had a higher correlation with pre-pregnancy BMI. Total body water, protein levels, mineral levels, bone mineral content, soft lean mass, fat-free mass, skeletal muscle mass, and the basal metabolic rate were protective factors for GDM after adjusting for some confounders. In all pregnant women, the waist-hip ratio was found to be up to 4.562 times the risk of GDM development, and gestational weight gain had the best predictive power for GDM. Gestational weight gain in early pregnancy, body fat mass, and the waist-hip ratio can assess the risk of GDM in pregnant women, which can allow clinicians to predict the occurrence of GDM in pregnant women as early as possible and implement interventions to reduce adverse perinatal outcomes.

## Introduction

Gestational diabetes mellitus (GDM) is diabetes diagnosed in the second or third trimester of pregnancy that was clearly overt not diabetes prior to gestation (1). GDM is an essential factor affecting maternal and infant health and is one of the most common complications during pregnancy (2). One study showed that the overall incidence of gestational diabetes has increased globally over the past decade (3). According to a 2018 meta-analysis, the prevalence of gestational diabetes in China ranged from 13.0% to 20.9% (4). GDM increases the risk of miscarriage, obstructed labor, and cesarean section in pregnant women, as well as the risk of perinatal macrosomia, fetal growth restriction, neonatal hypoglycemia, and even the risk of type 2 diabetes in children later in life (5). Patients with GDM also have an increased risk of developing diabetes and cardiovascular disease in later years period (6).

Obesity is one of the risk factors for GDM, especially visceral obesity in pregnant women (7). In a European study, the prevalence of GDM in obese women was reported to be close to 40% (8). Body mass index (BMI) is often used as a clinical measure of body fatness. Nevertheless, it does not distinguish between body fat content and nonfat content, e.g., muscular obesity is defined as an abundance of lean tissue mass with little body fat, such as in athletes; intangible obesity is defined as an excess of body fat, i.e., obesity (9). In the state of obesity, the human body stores too much energy in the form of fat, which leads to changes in some innate immune cells in adipose tissue, promotes the occurrence of adipose tissue inflammation, induces islet β-cell dysfunction, and eventually leads to systemic insulin resistance and glucose tolerance (9, 10). This obesity-induced insulin resistance can occur at all stages of life, including during pregnancy or the postpartum period. Myo-inositol, as a dietary supplement, can reduce insulin resistance (11), and myo-inositol supplementation in early pregnancy in overweight nonobese pregnant women can significantly reduce the incidence of GDM, which can contribute to the prevention and intervention of GDM in clinical practice (12). During pregnancy, to provide energy and nutrition to the fetus, maternal energy expenditure increases, and the intestinal tract has an increased ability to absorb fat, resulting in an increase in fat content in the mother’s body compared to that pre-pregnancy (13). However, excessive fat accumulation in the body and blood lipid disorders may lead to the development of diabetes (13). A study in 18 cities in China confirmed that pre-pregnancy overweight/obesity is a high-risk factor for the onset of GDM (14).

A bioelectric impedance assessment (BIA) is a simple and noninvasive method of assessing the body electrically. It provides a more accurate picture of the body’s muscle, fat, and bone mass and thus determines whether a person’s body composition is standard. However, a BIA cannot distinguish between maternal and fetal tissue (15, 16). It is a method for assessing the internal structure of a pregnant woman’s body in the early stages of pregnancy. It has become a routine perinatal examination to analyze the composition and proportions of body components from a microscopic point of view (17). There is a strong association between high fat content, low muscle mass, and the prevalence of diabetes mellitus (18). PBF reflects the percentage of thetotal body weight accounted for by the total body fat mass. At the same time, the visceral adiposity index (VAI) is a reliable indicator of the content of visceral adipose tissue (19). These indicators are reflected in the BIA examination, and the higher the fat content is, the greater the electrical impedance (20). The physical properties, measurement variables, and clinical significance of BIAs have been well described in many previously published reports (18), and their safety has been demonstrated in many studies in patients with renal disease, such as hemodialysis and transplant patients (15). Only a few domestic and international studies have explored the effect of body composition on GDM risk through BIAs. Moreover, body fat distribution varies with ethnicity, and study indicators and conclusions are primarily inconsistent (15, 21, 22). The influence of body composition during pregnancy on GDM risk was analyzed in a retrospective study of 22,223 pregnant women in southwest China. The visceral fat level, bone mineral content, and body fat percentage were significant predictors of GDM (23). In this study, multifrequency BIAs were used to determine the body composition of pregnant women in the first trimester to further explore the effect of body composition in the first trimester on GDM risk in different prepregnancy BMI groups in the Central Plains of China.

## Materials and methods

### Study design and patients

This study retrospectively analyzed pregnant women who visited the Third Affiliated Hospital of Zhengzhou University from January 1, 2021, to December 31, 2021, who were treated, had documented data, and received regular perinatal care. The inclusion criteria were as follows: (1) patients for whom a 75 g oral glucose tolerance test (OGTT) was performed at 24-28 weeks of gestation before body composition analysis; (2) patients aged ≥ 18 years old; (3) patients with a singleton pregnancy; and (4) patients who did not have diabetes before pregnancy. The exclusion criteria were (1) patients with pre-pregnancy cardiovascular disease, diabetes mellitus, and thyroid abnormalities; (2) patients with twin or multiple pregnancies; (3) patients with psychiatric disorders who were unable to complete the test; and (4) patients with missing data.

The above study was approved by the Ethics Committee of the Third Affiliated Hospital of Zhengzhou University, Henan Province (2022-143-01).

### Diagnostic criteria for GDM

According to the diagnostic criteria of the IADPSG 2010 (24), subjects underwent a 75 g-OGTT at 24-28 weeks of gestation, consumed a vegetarian diet while abstaining from meat, eggs, milk, and fruit the day before the OGTT, and fasted for 8-14 hours after dinner and the following morning (no later than 9 a.m.). Three hundred milliliters of liquid containing 75 g of glucose was taken orally within 5 minutes after drawing venous blood on an empty stomach. Venous blood was taken 1 h and 2 h after taking glucose (the time was counted from the time of drinking the glucose water), and plasma glucose was measured using the glucose oxidase method. The plasma glucose values while fasting and 1 h and 2 h after taking the glucose water were set at 5.1 mmol/L, 10.0 mmol/L, and 8.5 mmol/L, respectively. Pregnant women who met the diagnostic criteria for GDM were included in the GDM group, and those who did not were included in the normal group.

### Covariates

The general data of pregnant women in the first trimester (before 14 weeks of pregnancy) were retrospectively collected, including age, height, pre-pregnancy BMI, reproductive history, weight at the time of BIA, gestational age at the time of BIA, and gestational weight gain (GWG). Direct segmental multifrequency BIA (DSM-BIA method) was performed in the first trimester of pregnancy using an Inbody J30 device (instrument measurement frequencies 5 kHz, 50 kHz, 250 kHz). All data for body composition analysis were collected by trained nursing staff in the obstetric clinic in strict accordance with the instructions for use. Before the test, the pregnant woman was asked to empty her bladder, remove her coat, shoes, socks, accessories, and metal objects, and wipe her hands and feet with a wet paper towel. The measurement was taken while the patient was standing, with her heel flush with the foot electrode, her arm semibent and away from her body, and while grasping the handle of the device and placing her thumb on the oval electrode piece. The test lasted 30 seconds and the patient remained relaxed until the end of the test. As soon as the patient stepped off the device, the device automatically printed a standard report containing the following data: total body water (TBW), protein levels, mineral levels, bone mineral content (BMC), body fat mass (BFM), soft lean mass (SLM), fat-free mass (FMM), skeletal muscle mass (SMM), percent body fat (PBF), the waist-hip ratio (WHR), the visceral fat level (VFL), and the basal metabolic rate (BMR).

Pre-pregnancy BMI was calculated as follows: pre-pregnancy weight/height ^2^ (kg/m^2^). The pregnant women were classified by pre-pregnancy BMI according to the “WS/T428-2013 Adult Weight Determination” standard issued by the National Health and Family Planning Commission of the People’s Republic of China in 2013 (25). Pregnant women were grouped according to the pre-pregnancy BMI classification criteria: a BMI<18.50 kg/m^2^ was considered low weight before pregnancy, a BMI of 18.50-23.90 kg/m^2^ was considered normal weight before pregnancy, a BMI of 24.00-27.90 kg/m^2^ was considered overweight, and a BMI ≥28.00 kg/m^2^ was considered obese. The number of women with a pre-pregnancy BMI<18.50 kg/m^2^ and a BMI ≥28.00 kg/m^2^ was small in this study, so in the low-weight and normal groups, overweight and obese pregnant women were combined into one group for analysis. Gestational weight gain (GWG) was calculated by subtracting the reported pre-pregnancy weight from the recorded weight at the time of BIA (26).

Percent body fat was calculated as follows: fat mass/body mass × 100%. The basal metabolic rate was calculated as follows = 21.6 * fat-free mass (kg) + 370. The instrument used in this study classifies visceral fat mass on a scale of 1 to 30, which is expressed as the VFL, where 1 to 9 indicates a normal visceral fat mass, 10 to 14 indicates a high visceral fat mass, 15 to 29 indicates a high-fat content, and 30 indicates super high-fat content. A visceral fat grade of 10 is equivalent to 100 cm^2^ of visceral fat.

### Statistical analyses

SPSS 26.0 statistical software (International Business Machines Corporation, New York, United States of America) was used for data processing and analysis. The Kolmogorov−Smirnov test (K-S test) was used to analyze whether the data were normally distributed, which was expressed as (ᶍ̅ ± s) and compared between two groups using the two independent samples t test. Nonnormally distributed measurement data are expressed as medians (quartiles). Unordered categorical comparisons between groups were performed using the ᶍ^2^ test, and comparisons between two groups were performed using a two-independent sample nonparametric test (the Mann−Whitney U test). In the univariate analysis of body composition, the association with the risk of developing GDM was included in a multivariate analysis, using relative risks and 95% confidence intervals obtained from log-binomial regression and performing multivariate regression analysis using generalized linear regression. Linearity between continuous variables was assessed using Pearman’s correlation coefficient. A correlation heatmap was used to represent the correlation of continuous variables. The narrower the graph and the darker the color, the stronger the correlation. The area under the curve (AUC) was further calculated from the receiver operating characteristic (ROC) curve. The optimal cutoff value for each risk factor was calculated according to the Youden Index, which maximizes the following equation: J = maxc {Se(c) + Sp(c) 1}, where c is the cut-off point for the sum of Se (sensitivity) and Sp (specificity) to obtain the highest value (27). After selecting the cutoff point for each marker, the sensitivity and specificity at the best cutoff value were calculated. The Hosmer−Lemeshow test was used to assess the final model fit. P<0.05 was considered a statistically significant difference.

## Manuscript formatting

### Description of the overall pregnant women

**Figure 1:**
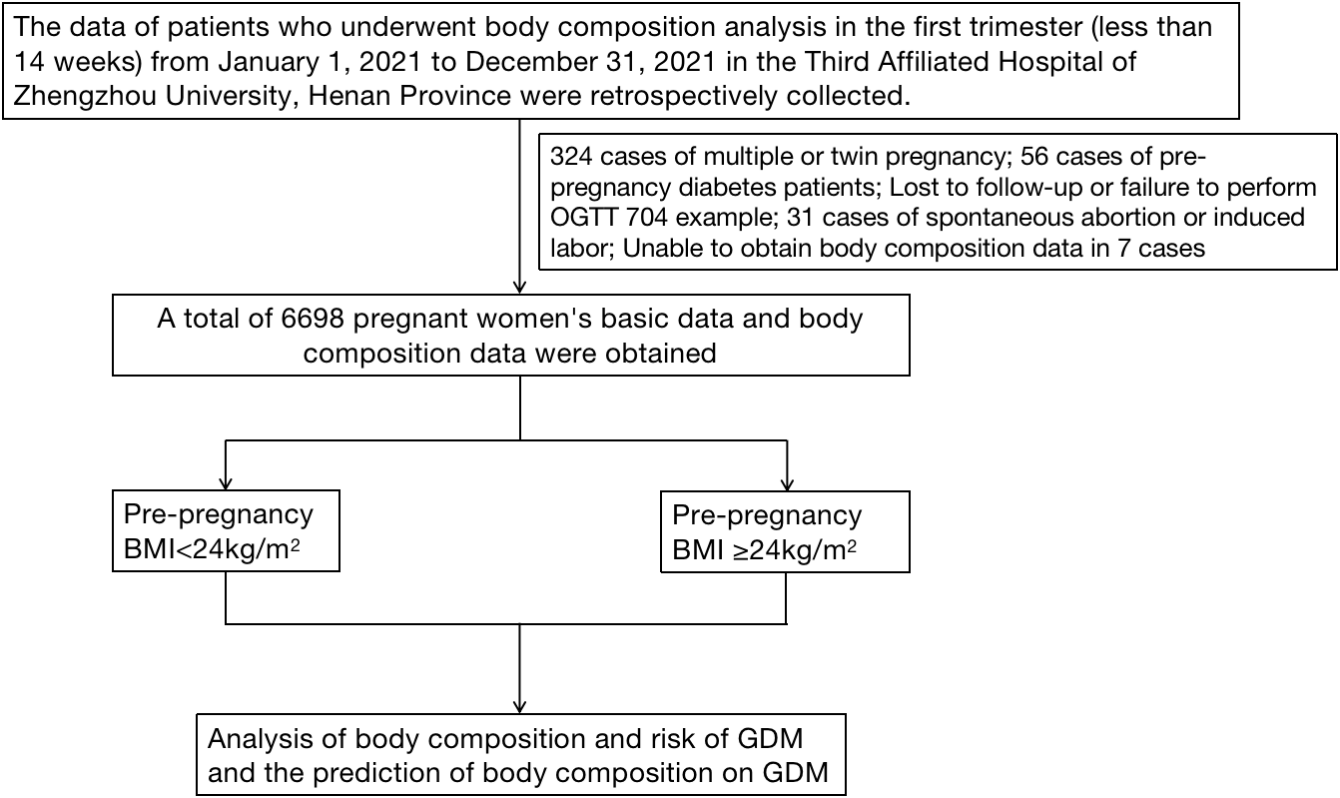
Technical route.

Initially, information was obtained for a total of 7820 pregnant women, including 324 women with twin or multiple pregnancies, 704 women with no OGTT results or missed visits, 31 women with spontaneous abortion or induced labor, 56 women with pre-pregnancy diabetes, and 7 women for whom BIA data were not available, resulting in 6698 pregnant women being included in the study (**Figure 1**). **Table 1** shows the essential characteristics of the 6698 pregnant women, including a total of 1109 women with GDM, with a detection rate of 16.56%. It was found that the age, gravidity, weight at the time of BIA, gestational age at the time of BIA, and GWG of the GDM group were higher than those of the normal group, but the height was lower than that of the normal group. The detection rate of GDM was higher in multiparous women. Regarding body composition, TBW, protein levels, mineral levels, BMC, BFM, SLM, FMM, SMM, PBF, WHR, VFL, and the BMR were all higher in the GDM group than in the normal group. There were significant differences (P<0.05).

**Table 1:** Basic characteristics of pregnant women.

General Features	Total (N = 6698)	GDM group (N = 1109)	Normal group (N = 5589)	t/z/ӽ^2^	*P*
Age (years)	30.20 ± 3.98	31.62 ± 4.07	29.92 ± 3.90	13.108	<0.001
Height (m)	1.61 ± 0.01	1.60 ± 0.06	1.61 ± 0.06	-3.344	0.001
Pre-pregnancy (Kg/m2)	23.41 ± 3.66	25.40 ± 4.19	23.02 ± 3.41	17.829	<0.001
Gravidity [M, (p25,p75)]	2 (1,2)	2 (1,3)	2 (1,2)	-4.689	<0.001
Parity [M, (p25,p75)]	0 (0,1)	0 (0,1)	0 (0,1)	-2.863	<0.001
Maternity history				7.264	0.007
multipara	2172 (32.43%)	398 (35.89%)	1774 (31.74%)		
primipara	4526 (67.57%)	711 (64.11%)	3815 (68.26%)		
Weight at the time of BIA (kg)	60.57 ± 9.88	65.36 ± 11.61	59.62 ± 9.21	15.518	<0.001
Gestational week at the time of BIA	12.15 ± 1.18	12.71 ± 1.58	12.04 ± 1.06	13.629	<0.001
GWG (Gestational weight gain,kg)	1.076 ± 0.36	1.41 ± 0.37	1.01 ± 0.32	34.087	<0.001
TBW (Total Body Water, kg)	29.37 ± 3.41	30.47 ± 3.83	29.16 ± 3.28	10.688	<0.001
Protein (kg)	7.79 ± 0.92	8.10 ± 1.02	7.73 ± 0.89	10.982	<0.001
Minerals (kg)	2.91 ± 0.35	3.00 ± 0.38	2.89 ± 0.38	9.118	<0.001
BMC (Bone Mineral Content, kg)	2.43 ± 0.29	2.50 ± 0.32	2.41 ± 0.28	8.992	<0.001
BFM (Body Fat Mass, kg)	20.49 ± 6.57	23.79 ± 7.69	19.84 ± 6.13	16.111	<0.001
SLM (Soft Lean Mass, kg)	37.65 ± 4.38	39.06 ± 4.91	37.37 ± 4.22	10.743	<0.001
FFM (Fat Free Mass, kg)	40.08 ± 4.66	41.57 ± 5.21	39.78 ± 4.48	10.667	<0.001
SMM (Skeletal Muscle Mass, kg)	21.52 ± 2.77	22.43 ± 3.08	21.35 ± 2.67	10.942	<0.001
PBF (Percent Body Fat, %)	33.18 ± 5.94	35.68 ± 5.88	32.67 ± 5.83	15.657	<0.001
WHR (Waist-Hip Ratio)	0.88 ± 0.05	0.90 ± 0.06	0.88 ± 0.05	13.616	<0.001
VFL (Visceral Fat Level)	9.43 ± 3.68	11.17 ± 3.94	9.08 ± 3.52	16.411	<0.001
BMR (Basal Metabolic Rate, kcal/day)	1228.50 ± 100.61	1267.91 ± 112.55	1229.29 ± 96.81	10.672	<0.001

### General information on pregnant women under different pre-pregnancy BMI groups

Under different pre-pregnancy BMI groupings, there were 4157 pregnant women with BMI <24 kg/m^2^, of which 456 (10.97%) were diagnosed with GDM; in a total of 2541 pregnant women with BMI ≥24 kg/m^2^, 653 (25.70%) were diagnosed with GDM.In the subgroup with pre-pregnancy BMI<24kg/m^2^ or in the subgroup with pre-pregnancy BMI<24kg/m^2^, the age, weight at the time of BIA, gestational age at the time of BIA, and GWG were all higher than those of the normal group, and there were statistically significant differences. See **Table 2** for details.

**Table 2:** Comparison of general data of pregnant women with different pre-pregnancy BMI.

	N	Age(years)	Height(m)	Gravidity [M, (p25,p75)]	Parity [M, (p25,p75)]	Weight at thetime of BIA(kg)	Gestational week at the time of BIA	GWG(kg)
pre-pregnancy BMI <24kg/m^2^								
GDM group	456	30.88 ± 3.94	1.61 ± 0.05	2(1,2)	0(0,1)	56.29 ± 5.56	12.75 ± 1.60	1.40 ± 0.39
Normal group	3701	29.48 ± 3.72	1.61 ± 0.05	1(1,2)	0(0,1)	54.95 ± 5.65	12.03 ± 1.07	1.00 ± 0.32
t/z		7.551	-1.615	-1.820	-0.457	4.573	9.332	21.167
P		<0.001	0.106	0.069	0.647	<0.001	<0.001	<0.001
pre-pregnancy BMI ≥24kg/m^2^								
GDM group	653	32.13 ± 4.09	1.60 ± 0.56	2(1,3)	0(0,1)	71.69 ± 10.47	12.69 ± 1.56	1.42 ± 0.36
Normal group	1888	30.79 ± 4.10	1.60 ± 0.56	2(1,3)	0(0,1)	68.76 ± 7.89	12.06 ± 1.03	1.02 ± 0.32
t		7.186	-0.633	-1.721	-0.596	6.532	9.579	25.492
P		<0.001	0.527	0.085	0.551	<0.001	<0.001	<0.001

### Analysis of body composition of pregnant women under different BMI groups

Statistical analysis showed that TBW, protein levels, mineral levels, BMC, BFM, SLM, FMM, SMM, PBF, the WHR, the VFL, and the BMR of pregnant women with a pre-pregnancy BMI < 24 kg/m^2^ and those with a pre-pregnancy BMI≥24 kg/m^2^ were higher in the GDM group than in the normal group. In the BMI<24 kg/m^2^, the BFM, PBF, WHR, and VFL of pregnant women in the GDM group were significantly different from those in the normal group (P<0.05). However, there was no significant difference in TBW, protein levels, mineral levels, BMC, SLM, FFM, SMM, or the BMR (P>0.05), as shown in **Table 3**.

**Table 3:** Analysis of body composition of pregnant women under different pre-pregnancy BMI.

	N	TBW(kg)	Protein(kg)	Minerals(kg)	BMC(kg)	BFM(kg)	SLM(kg)	FFM(kg)	SMM(kg)	PBF(%)	WHR	VFL	BMR(kcal/day)
Pre-pregnancy BMI <24kg/m^2^													
GDM group	456	28.31 ± 2.82	7.51 ± 0.75	2.80 ± 0.29	2.34 ± 0.24	17.66 ± 3.52	36.28 ± 3.62	38.63 ± 3.84	20.67 ± 2.27	31.22 ± 4.61	0.87 ± 0.04	7.89 ± 2.33	1204.38 ± 83.05
Normal group	3701	28.10 ± 2.75	7.44 ± 0.74	2.79 ± 0.29	2.33 ± 0.24	16.62 ± 3.53	36.00 ± 3.54	38.34 ± 3.76	20.48 ± 2.23	30.05 ± 4.65	0.86 ± 0.04	7.29 ± 2.19	1198.09 ± 81.25
t		1.523	1.894	0.771	0.967	5.946	1.579	1.542	1.789	5.080	5.086	5.268	1.556
P		0.128	0.058	0.441	0.334	<0.001	0.114	0.123	0.074	<0.001	<0.001	<0.001	0.120
Pre-pregnancy BMI ≥24kg/m^2^													
GDM group	653	31.98 ± 3.72	8.50 ± 0.99	3.14 ± 0.37	2.62 ± 0.31	28.06 ± 6.88	41.00 ± 4.76	43.63 ± 5.05	23.66 ± 2.97	38.80 ± 4.50	0.93 ± 0.05	13.47 ± 3.13	1312.28 ± 109.17
Normal group	1888	31.23 ± 3.24	8.30 ± 0.87	3.08 ± 0.34	2.57 ± 0.29	26.15 ± 5.16	40.03 ± 4.17	42.61 ± 4.43	23.05 ± 2.63	37.83 ± 4.25	0.91 ± 0.05	12.61 ± 2.93	1290.45 ± 95.71
t		2.462	4.585	3.443	3.231	6.512	4.612	4.544	4.613	4.935	5.223	6.142	4.541
P		<0.001	<0.001	0.001	0.001	<0.001	<0.001	<0.001	<0.001	<0.001	<0.001	<0.001	<0.001

TBW, total body water; BMC, bone mineral content; BFM, body fat mass; SLM, soft lean mass; FMM, fat free mass; SMM, skeletal muscle mass; PBF, percent body fat; WHR, waist-hip ratio; VFL, visceral fat level; BMR, basal metabolic rate.

### Correlation analysis of pre-pregnancy BMI and body composition

**Figure 2:**
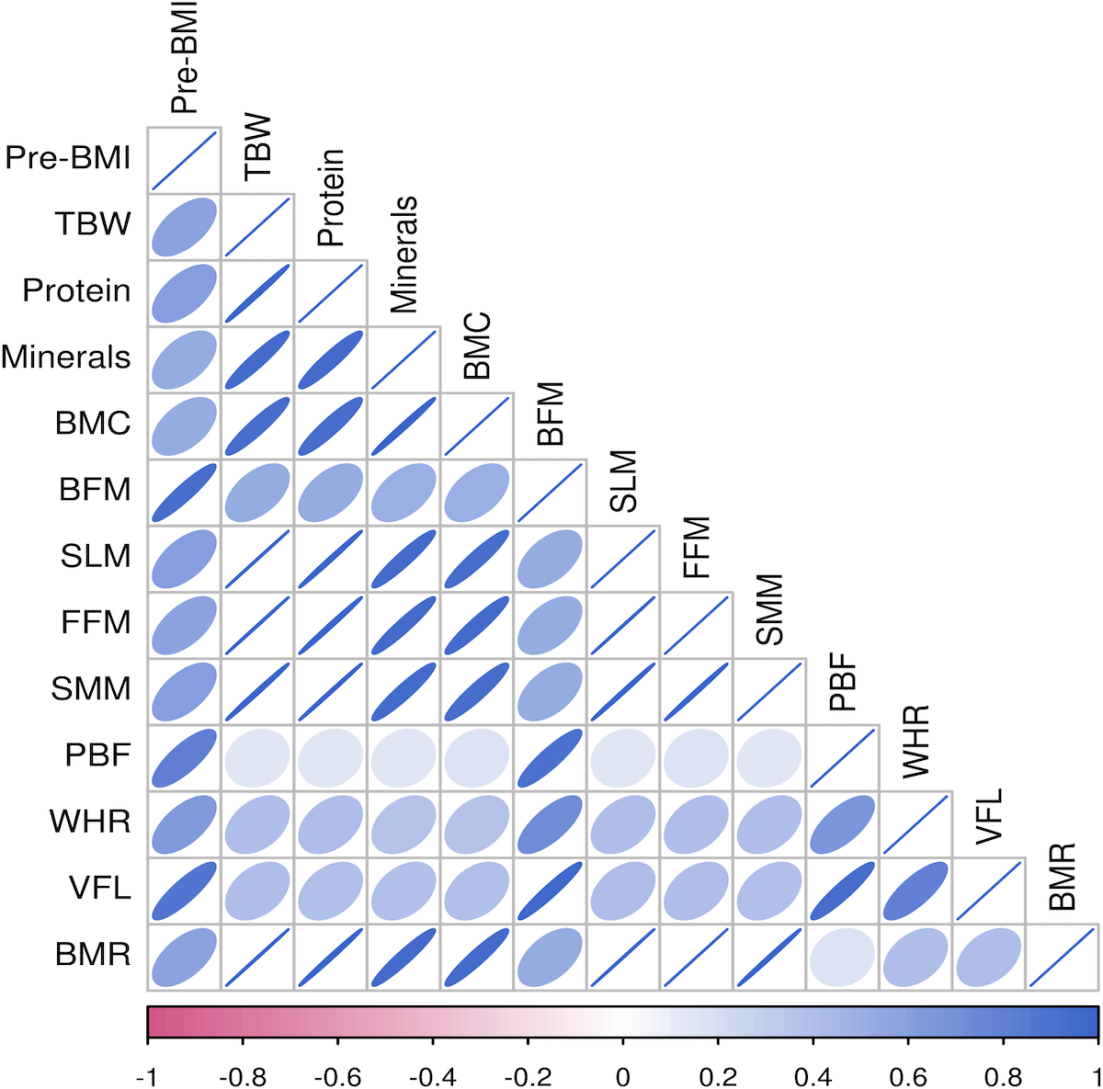
Heatmap of body composition correlations for pre-pregnancy BMI for all pregnant women.

**Figure 3:**
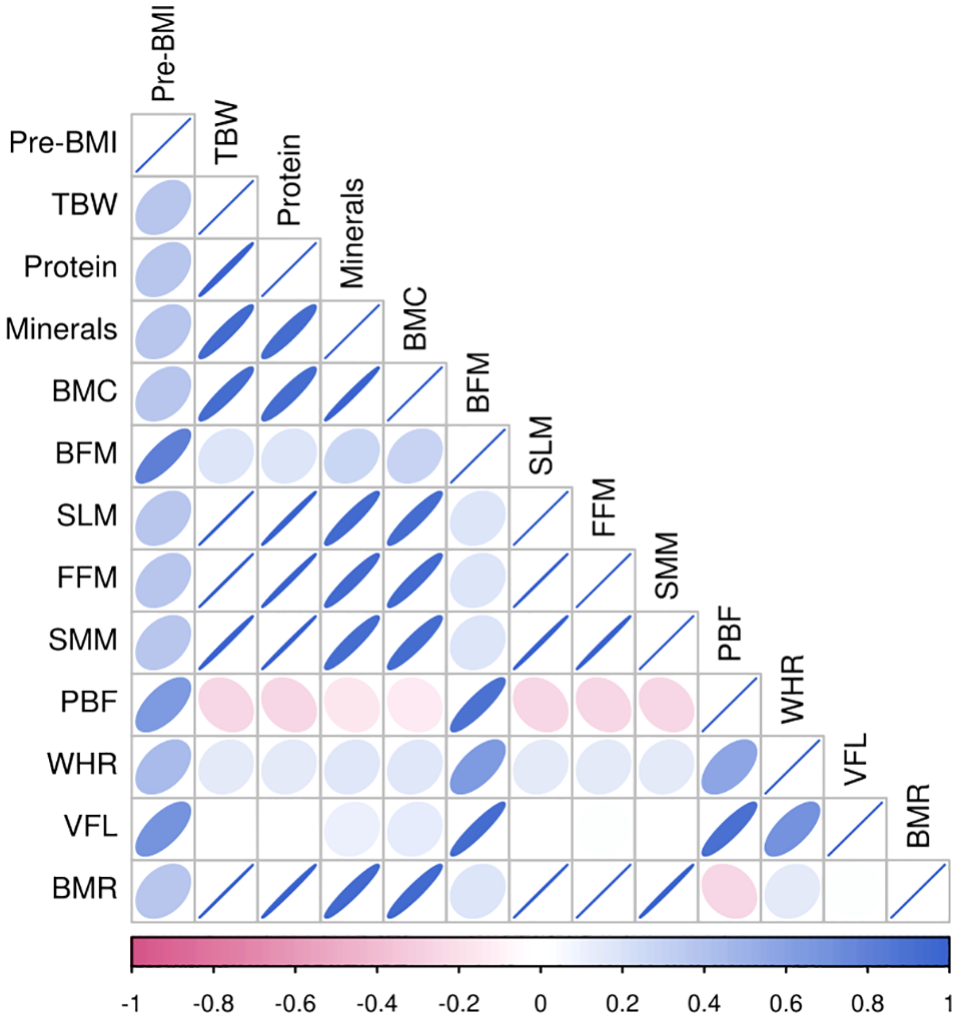
Heat map of body composition correlation in pregnant women with pre-pregnancy BMI < 24kg/m^2^.

**Figure 4:**
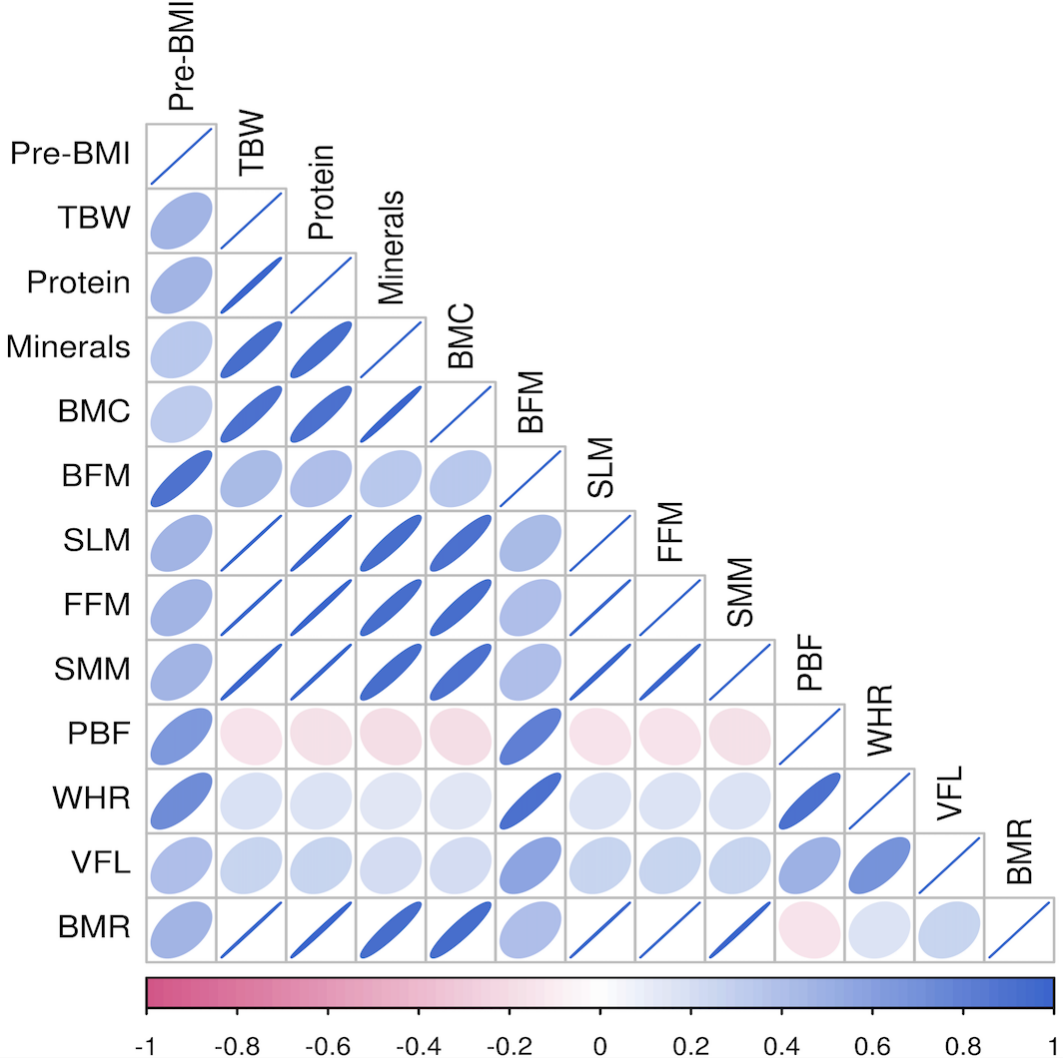
Heat map of body composition correlation in pregnant women with former BMI ≥ 24kg/m^2^.

Pre-pregnancy BMI was significantly positively correlated with TBW, protein levels, mineral levels, BMC, BFM, SLM, FMM, SMM, PBF, WHR, VFL, and the BMR (P<0.01) in the different groups. Among all pregnant women, the correlation between BMI before pregnancy and BFM was the strongest (r=0.953), followed by that between BMI before pregnancy and the VFL (r=0.873). Among women with a BMI<24 kg/m^2^ before pregnancy, BFM had the strongest correlation (r=0.812), followed by VFL (r=0.688). In women with a pre-pregnancy BMI≥24 kg/m^2^, BFM was strongly correlated with pre-pregnancy BMI (r=0.884), followed by WHR (r=0.732), as shown in **Figures 2**, **3**, **4**.

### Generalized linear regression of body composition and GDM risk

In the multivariate regression model, GDM was the dependent variable. The factors with statistically significant differences in the univariate analysis were included as the independent variables, and the multivariate generalized linear regression equation was analyzed. In the general information of pregnant women, the pre-pregnancy BMI, age, gestational weight gain (GWG) in the first trimester, and weight during BIA were all risk factors for GDM. This study found that GWG was related to a high risk of GDM. For every 1 kg increase in GWG, the risk of GDM increased by 4.08 times. This risk was higher in pregnant women with a BMI≥24 kg/m^2^ before pregnancy.

In the body composition index, each index item was positively correlated with the risk of GDM when no confounding factors were adjusted. In Model 3, TBW, protein levels, mineral levels, BMC, SLM, FFM, SMM, and the BMR were protective factors for GDM (P < 0.05). These protective factors were not found in the subgroup of pregnant women with a pre-pregnancy BMI≥24 kg/m^2^. After adjusting for confounding factors in Model 4, only the waist-hip ratio had a certain effect on GDM risk, and for each additional unit of the WHR, the risk of GDM increased by 4.562 times. Among pregnant women with a pre-pregnancy BMI <24 kg/m^2^, the body composition-related indicators in Model 2 were all related to the onset of GDM. In Model 3, TBW, SLM, FFM, and the BMR were negatively correlated with the incidence of GDM. In Model 4, mineral levels and BMC were protective factors for GDM, and WHR led to a higher risk of GDM occurrence. For pre-pregnancy BMI≥24 kg/m^2^, only in Model 1 and Model 2 was a positive correlation found between body composition and the onset of GDM. The WHR resulted in a higher risk of GDM onset during pregnancy. Body composition was not found to be associated with the risk of GDM in Model 3 or Model 4 after adjusting for confounding factors. See **Table 4** for details.

**Table 4:** Multivariate regression analysis of different pre-pregnancy BMI groups.

Index	ALL			Pre-pregnancy BMI<24kg/m^2^			Pre-pregnancy BMI ≥24kg/m^2^		
	RR	95% C I	P	RR	95% C I	P	RR	95% C I	P
Pre-pregnancy BMI	1.132	1.119~1.145	<0.001	1.209	1.146~1.275	<0.001	1.073	1.047~1.099	<0.001
Age(years)	1.089	1.076~1.102	<0.001	1.086	1.065~1.108	<0.001	1.058	1.043~1.073	<0.001
GWG(kg)	4.080	2.114~6.045	<0.001	3.689	3.346~4.032	<0.001	5.193	4.540~5.846	<0.001
Weight at the time of BIA(kg)	1.041	1.037~1.046	<0.001	1.038	1.022~1.053	<0.001	1.024	1.019~1.029	<0.001
TBW(kg)									
Model 1	1.089	1.072~1.106	<0.001	1.025	0.991~1.059	0.151	1.047	1.029~1.066	<0.001
Model 2	1.136	1.119~1.154	<0.001	1.079	1.032~1.129	0.001	1.092	1.067~1.117	<0.001
Model 3	0.962	0.940~0.984	0.001	0.951	0.911~0.993	0.021	0.981	0.955~1.009	0.183
Model 4	0.996	0.966~1.026	0.778	0.986	0.936~1.039	0.596	1.002	0.968~1.037	0.910
Protein(kg)									
Model 1	1.381	1.304~1.463	<0.001	1.119	0.989~1.265	0.074	1.187	1.095~1.285	<0.001
Model 2	1.596	1.506~1.692	<0.001	1.364	1.165~1.597	<0.001	1.367	1.255~1.489	<0.001
Model 3	0.886	0.813~0.965	0.006	0.882	0.754~1.032	0.116	0.933	0.844~1.032	0.180
Model 4	1.010	0.909~1.122	0.849	1.027	0.853~1.235	0.780	1.002	0.887~1.131	0.977
Minerals(kg)									
Model 1	1.961	1.701~2.261	<0.001	1.126	0.818~1.548	0.467	1.375	1.124~1.628	0.002
Model 2	2.377	1.751~3.266	<0.001	1.797	1.165~1.597	0.010	1.763	1.291~2.407	<0.001
Model 3	0.642	0.520~0.793	<0.001	0.385	0.252~0.586	0.500	0.811	0.636~1.035	0.092
Model 4	0.966	0.721~1.293	0.816	0.496	0.287~0.855	0.012	1.107	0.818~1.499	0.510
BMC(kg)									
Model 1	2.127	1.816~2.492	<0.001	1.193	0.816~1.745	0.362	1.413	1.120~1.784	0.004
Model 2	2.506	1.766~3.577	<0.001	2.284	1.323~3.944	0.003	1.803	1.266~2.567	0.001
Model 3	0.597	0.467~0.764	<0.001	0.337	0.205~0.554	0.383	0.776	0.584~1.032	0.082
Model 4	1.018	0.727~1.427	0.916	0.491	0.255~0.945	0.033	1.146	0.815~1.613	0.433
BFM(kg)									
Model 1	1.059	1.052~1.066	<0.001	1.078	1.050~1.107	<0.001	1.036	1.025~1.047	<0.001
Model 2	1.057	1.050~1.063	<0.001	1.076	1.048~1.105	<0.001	1.039	1.031~1.047	<0.001
Model 3	1.029	1.012~1.047	0.001	1.038	1.006~1.072	0.019	0.985	0.965~1.007	0.182
Model 4	1.002	0.980~1.024	0.849	1.010	0.972~1.050	0.603	0.998	0.973~1.024	0.890
SLM(kg)									
Model 1	1.069	1.056~1.082	<0.001	1.020	0.994~1.046	0.136	1.037	1.020~1.054	<0.001
Model 2	1.104	1.091~1.118	<0.001	1.062	1.026~1.100	0.001	1.070	1.052~1.090	<0.001
Model 3	0.971	0.953~0.989	0.001	0.963	0.932~0.996	0.028	0.985	0.965~1.007	0.182
Model 4	0.998	0.975~1.021	0.842	0.992	0.952~1.033	0.687	1.001	0.975~1.028	0.924
FFM(kg)									
Model 1	1.064	1.052~1.076	<0.001	1.018	0.994~1.043	0.146	1.034	1.018~1.050	<0.001
Model 2	1.099	1.086~1.111	<0.001	1.059	1.025~1.094	0.001	1.067	1.049~1.085	<0.001
Model 3	0.972	0.955~0.988	0.001	0.963	0.933~0.994	0.019	0.986	0.966~1.006	0.167
Model 4	0.998	0.976~1.020	0.849	0.990	0.952~1.029	0.603	1.002	0.977~1.027	0.890
SMM(kg)									
Model 1	1.113	1.092~1.134	<0.001	1.036	0.994~1.079	0.091	1.059	1.031~1.087	<0.001
Model 2	1.168	1.145~1.190	<0.001	1.104	1.047~1.164	<0.001	1.110	1.079~1.142	<0.001
Model 3	0.961	0.934~0.989	0.006	0.955	0.907~1.006	0.083	0.979	0.947~1.013	0.224
Model 4	1.004	0.969~1.040	0.824	1.002	0.942~1.066	0.994	1.004	0.964~1.046	0.840
PBF(%)									
Model 1	1.073	1.063~1.084	<0.001	1.051	1.030~1.072	<0.001	1.038	1.020~1.056	<0.001
Model 2	1.069	1.059~1.079	<0.001	1.046	1.025~1.067	<0.001	1.043	1.028~1.059	<0.001
Model 3	1.024	1.013~1.036	<0.001	1.021	1.003~1.039	0.019	1.010	0.996~1.025	0.168
Model 4	1.010	0.996~1.024	0.147	1.005	0.983~1.027	0.667	1.000	0.983~1.018	0.997
WHR									
Model 1	994.455	362.269~2729.849	<0.001	206.291	23.361~1821.689	<0.001	34.023	7.890~146.714	<0.001
Model 2	1051.493	421.458~2623.361	<0.001	272.083	31.447~2354.089	<0.001	67.603	18.049~253.205	<0.001
Model 3	4.342	1.427~13.218	0.010	13.374	2.031~88.062	0.007	1.816	0.502~6.576	0.363
Model 4	4.562	1.532~13.582	0.006	7.132	1.008~50.447	0.049	2.713	0.765~9.620	0.122
VFL									
Model 1	1.121	1.105~1.138	<0.001	1.110	1.067~1.155	<0.001	1.071	1.045~1.098	<0.001
Model 2	1.113	1.099~1.127	<0.001	1.103	1.062~1.146	<0.001	1.077	1.055~1.099	<0.001
Model 3	1.038	1.016~1.059	0.001	1.030	0.991~1.071	0.129	1.014	0.989~1.041	0.280
Model 4	1.014	0.991~1.037	0.238	0.997	0.955~1.042	0.906	1.006	0.979~1.033	0.664
BMR(kcal/day)									
Model 1	1.003	1.002~1.003	<0.001	1.001	1.000~1.002	0.142	1.002	1.001~1.002	<0.001
Model 2	1.004	1.004~1.005	<0.001	1.003	1.001~1.004	0.001	1.003	1.002~1.004	<0.001
Model 3	0.999	0.998~0.999	0.001	0.998	0.997~1.000	0.020	0.999	0.998~1.000	0.165
Model 4	1.000	0.999~1.001	0.854	1.000	0.998~1.001	0.622	1.000	0.999~1.001	0.898

RR, relative risk; CI, confidence interval.

Model 1, without adjusting for confounding factors; Model 2, adjusted for age(years), height(kg), gravidity, parity; Model 3, adjusted for weight at the time of BIA(kg), gestational week at the time of BIA, GWG; Model 4, adjusted for Model 2 +Model 3.

### Predictive value of general data and body composition for GDM under different pre-pregnancy BMI groups

The predictive value for GDM was analyzed based on the general data of pregnant women and the related body composition indicators. In the results for body composition, the area under the ROC curve of BFM for predicting GDM in all pregnant women was larger (0.663), the 95% CI was 0.645-0.680, the Youden index was 0.252, and the best cutoff value was 20.95; for the VFL, the area under the curve was 0.656, the 95% CI was 0.639-0.674, the Youden index was 0.236, and the optimal cutoff value was 10.5. Among pregnant women with a pre-pregnancy BMI <24 kg/m^2^, the area under the ROC curve of BFM for predicting GDM was the largest (0.584), the 95% CI was 0.556-0.612, and the Youden index was 0.120; for PBF and the VFL, the area under the curve for both was 0.577, the Youden index was 0.118 and 0.117, respectively, and the optimal cutoff values were 32.65 and 8.5, respectively. Among pregnant women with a pre-pregnancy BMI ≥24 kg/m^2^, the area under the ROC curve of BFM for predicting GDM was the largest (0.584), the 95% CI was 0.558-0.609, the Youden index was 0.143, and the best cutoff value was 28.85; for the VFL, the area under the curve was 0.656, the 95% CI was 0.553~0.604, the Youden index was 0.121, and the optimal cutoff value was 13.5; (see **Tables 5**, **6** for details). The results will only be reproduced in a Chinese population using the same equipment.

**Table 5:** Analysis of the predictive effect of general indicators under pre-pregnancy BMI on GDM.

	Classification	AUC	P	95%CI	Cutoff points	Sensitivity	Specificity	Youden index
Pre-pregnancy BMI	All	0.675	<0.001	0.657~0.692	23.43	0.665	0.606	0.271
	Pre-pregnancy BMI <24kg/m^2^	0.600	<0.001	0.573~0.628	21.71	0.564	0.591	0.155
	Pre-pregnancy BMI ≥24kg/m^2^	0.609	<0.001	0.583~0.634	27.53	0.478	0.715	0.193
Age(years)	All	0.617	<0.001	0.599~0.635	29.5	0.703	0.464	0.167
	Pre-pregnancy BMI <24kg/m^2^	0.600	<0.001	0.573~0.626	29.5	0.651	0.509	0.160
	Pre-pregnancy BMI ≥24kg/m^2^	0.591	<0.001	0.566~0.616	31.5	0.534	0.593	0.117
GWG(kg)	All	0.795	<0.001	0.779~0.812	1.415	0.564	0.921	0.485
	Pre-pregnancy BMI <24kg/m^2^	0.788	<0.001	0.762~0.815	1.415	0.561	0.924	0.485
	Pre-pregnancy BMI ≥24kg/m^2^	0.796	<0.001	0.774~0.817	1.415	0.567	0.915	0.482
Weight at the time of BIA(kg),	All	0.653	<0.001	0.635~0.671	62.25	0.564	0.662	0.226
	Pre-pregnancy BMI <24kg/m^2^	0.564	<0.001	0.537~0.591	55.05	0.594	0.510	0.104
	Pre-pregnancy BMI ≥24kg/m^2^	0.584	<0.001	0.557~0.610	73.05	0.391	0.758	0.149

**Table 6:** Analysis of the predictive effect of different pre-pregnancy BMI lower body composition indexes on GDM.

	Classification	AUC	P	95%CI	Cutoff points	Sensitivity	Specificity	Youden index
TBW(kg)	All	0.599	<0.001	0.580~0.617	39.65	0.019	0.996	0.015
	Pre-pregnancy BMI <24kg/m^2^	0.514	0.332	0.486~0.542	30.55	0.219	0.815	0.034
	Pre-pregnancy BMI ≥24kg/m^2^	0.554	<0.001	0.529~0.580	32.05	0.371	0.629	0.009
Protein(kg)	ALL	0.601	<0.001	0.583~0.620	8.15	0.454	0.696	0.15
	pre-pregnancy BMI <24kg/m2	0.518	0.202	0.490~0.546	8.05	0.239	0.794	0.033
	pre-pregnancy BMI ≥24kg/m2	0.555	<0.001	0.529~0.581	8.55	0.455	0.629	0.084
Minerals(kg)	All	0.585	<0.001	0.566~0.604	3.005	0.454	0.670	0.124
	Pre-pregnancy BMI <24kg/m^2^	0.505	0.722	0.477~0.533	3.115	0.145	0.877	0.022
	Pre-pregnancy BMI ≥24kg/m^2^	0.541	0.002	0.515~0.567	3.225	0.391	0.677	0.068
BMC(kg)	All	0.581	<0.001	0.563~0.600	2.515	0.454	0.664	0.118
	Pre-pregnancy BMI <24kg/m^2^	0.507	0.619	0.479~0.535	2.015	0.936	0.086	0.022
	Pre-pregnancy BMI ≥24kg/m^2^	0.537	0.005	0.511~0.563	2.875	0.190	0.875	0.065
BFM(kg)	All	0.663	<0.001	0.645~0.680	20.95	0.626	0.626	0.252
	Pre-pregnancy BMI <24kg/m^2^	0.584	<0.001	0.556~0.612	16.35	0.662	0.458	0.120
	Pre-pregnancy BMI ≥24kg/m^2^	0.584	<0.001	0.558~0.609	28.85	0.377	0.766	0.143
SLM (kg)	All	0.599	<0.001	0.581~0.618	39.45	0.442	0.707	0.149
	Pre-pregnancy BMI <24kg/m^2^	0.515	0.306	0.486~0.543	38.95	0.237	0.798	0.035
	Pre-pregnancy BMI ≥24kg/m^2^	0.554	<0.001	0.529~0.580	40.25	0.541	0.547	0.088
FFM(kg)	All	0.598	<0.001	0.581~0.617	41.15	0.507	0.641	0.148
	Pre-pregnancy BMI <24kg/m^2^	0.514	0.325	0.486~0.542	34.55	0.877	0.161	0.038
	Pre-pregnancy BMI ≥24kg/m^2^	0.553	<0.001	0.528~0.579	44.05	0.429	0.658	0.087
SMM (kg)	All	0.601	<0.001	0.583~0.620	22.65	0.458	0.692	0.152
	Pre-pregnancy BMI <24kg/m^2^	0.517	0.224	0.489~0.546	22.55	0.213	0.822	0.035
	Pre-pregnancy BMI ≥24kg/m^2^	0.555	<0.001	0.529~0.581	24.25	0.375	0.714	0.089
PBF(%)	All	0.647	<0.001	0.629~0.665	33.75	0.657	0.568	0.225
	Pre-pregnancy BMI <24kg/m^2^	0.577	<0.001	0.549~0.605	32.65	0.423	0.695	0.118
	Pre-pregnancy BMI ≥24kg/m^2^	0.563	<0.001	0.537~0.588	36.95	0.669	0.447	0.116
WHR	All	0.632	<0.001	0.614~0.651	0.885	0.609	0.587	0.196
	Pre-pregnancy BMI <24kg/m^2^	0.570	<0.001	0.542~0.598	0.855	0.636	0.468	0.104
	Pre-pregnancy BMI ≥24kg/m^2^	0.569	<0.001	0.543~0.595	0.945	0.354	0.762	0.116
VFL	All	0.656	<0.001	0.639~0.674	10.5	0.551	0.685	0.236
	Pre-pregnancy BMI <24kg/m^2^	0.577	<0.001	0.549~0.605	8.5	0.401	0.716	0.117
	Pre-pregnancy BMI ≥24kg/m^2^	0.579	<0.001	0.553~0.604	13.5	0.466	0.655	0.121
BMR(kcal/day)	All	0.598	<0.001	0.580~0.617	1259.5	0.505	0.643	0.148
	Pre-pregnancy BMI <24kg/m^2^	0.514	0.319	0.486~0.543	1116.5	0.875	0.162	0.037
	Pre-pregnancy BMI ≥24kg/m^2^	0.553	<0.001	0.527~0.579	1300.5	0.521	0.567	0.088

AUC, area under curve.

## Discussion

In this study, the generalized linear regression model found that in all groups of pregnant women, pre-pregnancy BMI, age, gestational weight gain in the first trimester, and weight at the time of BIA were all risk factors for the onset of GDM. However, gestational weight gain in the first trimester was positively correlated with the risk of GDM.

In the body composition analysis, the body composition indicators were all positively correlated with the risk of GDM in Model 1; in Model 3, TBW, protein levels, mineral levels, BMC, SLM, FMM, SMM, and the BMR were protective factors against GDM. After Model 4 was adjusted for confounders, only WHR was positively associated with the occurrence of GDM. Among pregnant women with a pre-pregnancy BMI <24 kg/m^2^, the body composition-related indicators in Model 2 were all associated with the onset of GDM. In Model 3, TBW, SLM, FMM, and the BMR were negatively correlated with GDM onset. In Model 4, mineral levels and BMC were protective factors against GDM. The WHR has a higher risk of GDM. Only in Model 1 and Model 2 was a prepregnancy BMI≥24 kg/m^2^ found to be positively correlated with the onset of GDM, and there were no protective factors. In the prediction model, gestational weight gain in the first trimester had a higher predictive value for GDM, followed by pre-pregnancy BMI. Among the body composition indicators, BFM and PBF had higher predictive value for GDM in all groups of pregnant women. According to the pre-pregnancy BMI groups, the predictive risk value of body composition-related indicators for GDM needs further investigation.

With lifestyle changes, the incidence of GDM is increasing yearly, and it has become a significant public health problem in China (3). BMI is a crude marker of obesity that reflects current nutritional status but does not provide information on fat distribution. BIAs provide a more detailed assessment of body composition and compensates for the deficiencies associated with BMI (16). Previous studies have shown that BIAs are better predictors of pregnancy and postpartum outcomes than BMI (16). However, this study found that the predictive value of BMI and GWG before pregnancy in all the included pregnant women was higher than that of body composition detected by BIA, and body composition-related indicators in the first trimester had a specific predictive effect on the incidence of GDM. Pre-pregnancy BMI reflects the basic nutritional levels of women, which are closely related to the health status of the mother and fetus after pregnancy (28). A high pre-pregnancy BMI increases the risk of adverse pregnancy outcomes such as GDM, cesarean section, macrosomia, and postpartum hemorrhage (29). Pregnant women with a low BMI before pregnancy have insufficient fat reserves, poor nutritional levels, and reduced micronutrients, which can lead to iron deficiency and anemia during pregnancy (30). The pre-pregnancy BMI, as a controllable factor, suggests that women with a high BMI at the time of pregnancy should have a balanced diet, increase their amount of exercise and avoid overnutrition to reduce their body mass and reach the standard weight level as much as possible. At the same time, it is recommended that maternal and child health care institutions and hospital obstetrics and gynecology departments increase the popularization and publicity of the reasonable range of pre-pregnancy BMI for women preparing for pregnancy and scientifically guide dietary habits and lifestyles for women with a high pre-pregnancy BMI to help them reduce their GDM risk.

GWG is closely related to the short-term and long-term health of mothers and babies. Excessive weight gain during pregnancy is associated with gestational hypertension, GDM, postpartum obesity, and even long-term hypertension, diabetes, and metabolic syndrome (29, 31). In this study, it was found that the GDM group gained weight faster in early pregnancy than the normal group, and GWG in the first trimester had a strong predictive ability for GDM. This study showed that weight gain in early pregnancy is closely related to GDM. Excessive weight gain in early pregnancy increases the risk of GDM by 4.080 times and is an independent risk factor for GDM. A pre-pregnancy BMI ≥24 kg/m^2^ indicated an increased weight of pregnant women in the first trimester, which increased the risk of GDM by 5.193 times. People who are overweight or obese before pregnancy may have metabolic disorders before pregnancy. Weight gain in early pregnancy further worsens metabolic disorders, strengthens insulin resistance and increases the incidence of GDM (32). During the COVID-19 lockdown, lifestyle habits and eating patterns were affected, and outdoor activities were severely restricted. For pregnant women with GDM, weight gain during the lockdown period led to a higher BMI at delivery (33). The incidence of GDM increased during the time interval associated with the COVID-19 lockdown and in the following months (34). Therefore, paying attention to weight gain in early pregnancy and providing individualized medical nutrition therapy for patients who gain more weight in early pregnancy can reduce weight gain during pregnancy, reduce the rate of poor weight control, effectively control blood glucose and lipid levels, and reduce the incidence of maternal and infant adverse outcomes.

Different human body components have essential functions. Human body components are composed of water, protein, fat, inorganic salts, and other substances, and their proportions can reflect the nutritional status of the body to a specific extent (35). The correlation heatmap of this study showed that there was a certain correlation between body composition indicators and pre-pregnancy BMI. Staelens et al. found that the total water content was significantly increased during pregnancy (36). In this study, it was found that the TBW of pregnant women in the GDM group was higher than that in the normal group. Pregnant women with GDM may be in a hyperglycemic state for a long time, with immense osmotic pressure, and increased vascular permeability, so the extracellular water increases accordingly. This suggests that women with GDM may have problems with polyhydramnios (37).

Protein is an essential nutrient for the health of the mother and fetus and a regulator of glucose metabolism (38). Bao proposed that high protein intake before pregnancy increases the risk of GDM (39). Insufficient protein intake during pregnancy can lead to poor fetal development, miscarriage, deformities, etc., and it is not easy for these mothers to recover after delivery (40). Dietary protein intake can reduce blood glucose levels in the body by stimulating insulin secretion, thereby affecting the blood glucose status of the body (41). Inadequate protein intake during pregnancy will lead to insufficient metabolic substrates such as amino acids, thereby affecting maternal and infant outcomes. Therefore, pregnant women should pay attention to the lack of various body components and ensure the intake of an appropriate amount of high-quality protein every day.

Minerals have the functions of maintaining cell osmotic pressure, acid-base balance, and muscle excitability (42). In different pregnancy periods, due to the other conditions of maternal weight gain, maternal tissue growth, and fetal growth, pregnant women have additional requirements for various minerals (43). Due to the physiological changes, plasma volume, and glomerular filtration rate during each pregnancy, the mineral content in plasma decreases gradually with the progression of pregnancy (44). The lack and excess of minerals can directly affect the growth and development of the fetus in pregnant women, leading to different degrees of dysfunction in pregnant women and causing miscarriage and fetal birth defects (45). Therefore, attention should be given to mineral supplementation during pregnancy, even before pregnancy. Optimal mineral supplementation can significantly reduce various pregnancy complications (46) and ensure the health of the mother and the normal development of the fetus. Currently, there is no research on the relationship between in pregnant women’s body composition mineral levels and GDM risk. In this study, it was found that mineral levels had a low ability to predict the risk of GDM, and more prospective studies are needed to discuss this issue.

BMC refers to inorganic salts that make up bones and maintain bone density, in which calcium is the main component (23). The increased calcium demand during pregnancy is mainly used for the mineralization of fetal bones, so the lack of calcium in infants will lead to growth delay and bone deformation (46). Pregnant women have different degrees of calcium loss, which is evident in the third trimester of pregnancy (47). Increasing calcium intake and participating in outdoor activities during pregnancy can not only prevent bone loss in pregnant women but also ensure the normal development of the fetus (48). Zhang’s research first found that bone minerals in the first trimester of pregnancy are significant risk factors for GDM (23). However, our research found that BMC was a protective factor against GDM in Model 3. The research showed that there was no difference in bone mineral content between the GDM group and the non-GDM group in early pregnancy (49), which is contrary to our research results. Therefore, BMC during pregnancy needs to be further assessed with larger sample sizes. In this study, it was found that the TBW, protein levels, mineral levels, and BMC of the pregnant women in the GDM group were higher than those in the normal group. This is consistent with Moreno’s findings (50). Women with GDM have higher body weight during pregnancy, so various body components during pregnancy are also relatively increased.

SLM is determined by the addition of TBW and proteins in the body and is made up of skeletal and smooth muscle (51). Women with type 1 diabetes have lower total lean body mass and significantly less muscle area (52, 53). SMM plays a significant role in glucose homeostasis. Low skeletal muscle mass increases insulin resistance and diabetes risk (54). Maintaining the functional level of skeletal muscle is vital in maternal and fetal health. Pregnant women are faced with a reduction in skeletal muscle content caused by factors such as decreased activity and unbalanced dietary nutrition, and the risk of metabolic abnormalities caused by these factors is also worthy of attention (55). In this study, it was found that the SMM of the GDM group was higher than that of the normal group. Shin proposed that overweight women have more muscle mass, but this excess muscle mass is considered metabolically inactive because these women have insulin resistance (54). There are few studies on the correlation between skeletal muscle function indices and glucose and lipid metabolism during pregnancy. This study also found that SLM and SMM were protective factors for GDM, but their predictive risk value for GDM was not high. Therefore, further analysis of SLM and SMM with a larger sample size is required in future studies, taking the effects of physical activity and sedentary time into account.

The total body water, protein level, and muscle overlap is called FFM. FFM is a critical determinant of resting energy expenditure during pregnancy (56). Studies have reported that water and electrolytes in the human body are highly correlated with fat-free content, and 50 kHz whole-body BIA measurements are often used in conjunction with anthropometry to predict FFM (57). This study found that in pregnant women with a pre-pregnancy BMI <24 kg/m^2^, when weight at the time of BIA, gestational age at the time of BIA, and GWG were included as confounding factors, it was also found that FFM and SMM were negatively correlated with the incidence of GDM. This may be related to the fact that a high FFM may be connected to endogenous glucose output and contribute to blood glucose control (56). In a prospective cohort study in China, Wang et al. found a positive relationship between FFM and birth weight, and a woman had a FFM ≥ 40.76 kg, the risk of a birth weight over 4 kg was significantly increased by 2.47-fold (58). This may be related to pre-pregnancy obesity status, rapid fetal growth during pregnancy, and an increased TBW in the third trimester.

Adipose tissue is not only a storage area for energy but also an organ for releasing endocrine and immune signals. Therefore, the excessive accumulation of adipose tissue can affect the normal physiological functions of the body (15). After pregnancy, the intake of nutrients and calories gradually increases, the amount of exercise relatively decreases, fat accumulates, and the body fat percentage rises without any significant increase in activity (59). In our study, we found that women in the GDM group had significantly higher body fat mass (BFM) and percent body fat (PBF) than women in the normal group. A multifactorial analysis found that BFM and PBF were independent risk factors for the development of GDM (P<0.05), which is consistent with the findings of many studies (21, 22, 60). In Sommer’s reflection in a multiethnic population, it was found that the increase in BMI and BFM was positively correlated with GDM, and an increase in BMI of 0.21 kg/week was associated with a 1.23-fold increase in the risk of GDM (22). Some studies have shown that PBF is a better predictor of GDM than BMI (59). Zhao scholars suggested that the higher risk of diabetes in the high PBF group among those with normal BMI may be related to their low insulin sensitivity index (61). Liu et al. mentioned that pregnant women with a PBF higher than 28% had a higher risk of GDM than those with a normal PBF (21). A prospective cohort study by Qing found that BFM did not change significantly in the first trimester. At the same time, body weight (BW) and BFM increased in the second trimester and were positively correlated with GDM risk (56). Some scholars have also proposed that the increase in PBF before pregnancy also impacts GDM risk (62). In this study, it was found that the BFM and PBF of the pregnant women in the GDM group were relatively higher than those of pregnant women in the normal group, which is basically consistent with previous studies. For overweight/obese pregnant women, detecting their body fat distribution and identifying metabolically healthy obesity and metabolically abnormal obesity are helpful for the early detection of GDM high-risk groups. Therefore, we must manage pregnant women with an increased pre-pregnancy BMI and abnormal BFM or PBF.

Most of the body’s adipose tissue is located subcutaneously, while a small amount of adipose tissue accumulates in the abdomen (63). Subcutaneous fat has been reported to increase leptin and tumor necrosis factor alpha (TNF-α) secretion and decrease insulin sensitivity, while visceral fat can increase insulin resistance (56). Asian populations have more abdominal and visceral fat than European populations in China and South Asia (64). The excessive accumulation of abdominal fat can increase serum inflammatory factor levels, induce a chronic inflammatory response, reduce insulin sensitivity and affect pancreatic β-cell function, which in turn can lead to disorders of glucolipid metabolism (65). A mild inflammatory response is already present in normal pregnancy (7), and inflammatory factors are involved in insulin resistance and even GDM through different pathways in the body. Adipose tissue secretes many adipokines and cytokines. For example, lipocalin is positively associated with insulin sensitivity, and TNF-α and interleukin-6 (IL-6) activate the inflammatory response, thus creating a vicious cycle. The proinflammatory state of the body in GDM patients may also be associated with future type 2 diabetes and cardiovascular disease (66). Visceral fat is commonly used to describe intra-abdominal fat, including intraperitoneal fat (mesenteric and omental fat) and retroperitoneal fat, with the former flowing directly into the portal circulation and the latter into the systemic circulation. Excess visceral fat is also referred to as central or centripetal obesity (67). Excess visceral fat produces high levels of free fatty acids, increasing hepatic glycogen isogenesis and glycogenolysis, and is strongly associated with insulin resistance (68). Kim found through a cohort study that a higher visceral fat area (VFA) was an independent risk factor for type 2 diabetes (69). This study found that the VFL of pregnant women in the GDM group was significantly higher than that in the control group. Further multivariate analysis found that the VFL of pregnant women in early pregnancy was positively correlated with the incidence of GDM and had a specific predictive value for the occurrence of GDM. Zhang et al. mentioned in their study that VFL was closely associated with increased fasting glucose and HbA1c levels in GDM patients. HbA1c was closely related to elevated GDM risk and could be a risk factor for GDM (23).

Waist circumference (WC) is the body circumference at the abdominal level, which is a simple and effective indicator for evaluating central fat and has a significant predictive value in the risk of human metabolic diseases, such as hypertension, coronary heart disease, diabetes, and blood lipid disorders (70). In a Brazilian analysis of 5251 women with WC measurements at mid-gestation, it was found that a WC over 82 cm had a sensitivity of 63% and a specificity of 57% in predicting GDM (71). In a prospective cohort study conducted in China, BMI and WC were found to be associated with the development of GDM in Chinese pregnant women in early pregnancy, with a dramatic increase in the risk of GDM when the WC was ≥78.5 cm (72). The hip circumference is the horizontal perimeter of the most protruding part of the buttocks, which reflects the development of hip bones and muscles. It is also an adequate measure of hip fat (73). Snijder MB et al. prospectively found that a large hip circumference effectively reduced the risk of type II diabetes (74). The WHR is the ratio of the WC to hip fat, another critical index used to determine central obesity. In exploring the WHR as a predictor of GDM in Asian Indians, Madhavan et al. found that a high WHR was associated with an increased risk of GDM and was associated with an increased risk of GDM; the prevalence of GDM was seven times higher in the high WHR group than in the low WHR group (WHR ≤ 0.85) (73). In this study, the WHR was found to be an independent risk factor for GDM and had a particular predictive value for GDM. Basraon also suggested that the value of the WHR in predicting GDM is comparable to that of BMI [AUC: 0.68 (BMI), 0.63 (WHR)] (75).

The BMR is the most basic energy consumption to maintain the body’s life activities. Body composition changes dynamically during energy consumption. A reasonable BMR is significant for recommending dietary energy consumption during pregnancy (76). Pregnancy is a unique and complex physiological process. Due to the physiological needs of pregnancy, the body composition and the BMR of women change after pregnancy. Studies have shown that the BMR in the third trimester will increase by approximately 11% compared with that in the first trimester (77). The extra energy intake during pregnancy increases the body fat composition of pregnant women, and excessive body fat storage during pregnancy can lead to maternal obesity and other health problems (78). Under the guidance of body composition monitoring, an average body weight and body fat can be maintained, and the increase in body fat during pregnancy can be controlled to keep the body composition of pregnant women within a reasonable range. The results showed that the BMR of overweight/obese pregnant women before pregnancy was significantly higher than that of women with normal BMI before pregnancy. Therefore, a reasonable basal metabolic value and body composition status are of great significance for nutrition education before and during pregnancy and for recommending dietary energy consumption during pregnancy.

The advantage of this study is that pre-pregnancy BMI was used to group and analyze pregnant women to predict the risk of GDM. There is no such analysis at present. Medical staff should attach great importance to women with an abnormal pre-pregnancy BMI, improve pregnant women’s awareness of weight control before pregnancy, and provide them with personalized guidance as soon as possible to formulate a reasonable range of weight gain. This study lacks pre-pregnancy body composition measurement data, and it is difficult to see the variation range of body composition-related indicators from pre-pregnancy to early pregnancy. Changes in body composition during pregnancy also impact pregnancy outcomes, which needs further research. Because body fat distribution is influenced by age, ethnicity, physical activity level, and total fat mass, there are differences in body composition distributions. Therefore, the index conclusions of the best GDM prediction methods in different countries and regions are still controversial. There was no further stratified analysis of age in this study, and we will gradually supplement samples in future research to further explore the influence of various factors on body composition and pregnancy outcomes.

## Conclusion

In conclusion, regardless of the pre-pregnancy BMI level, all indicators of the BIA were independently related to the risk of GDM. Further analysis of the ROC curve showed that the body composition indicators of pregnant women in the first trimester had a particular predictive value for GDM. This study also found that excessive weight gain in the first trimester for GDM patients has a substantial predictive value for GDM. This suggests that medical staff should attach great importance to women with an abnormal pre-pregnancy BMI, improve pregnant women’s awareness of weight control before pregnancy, and provide them with personalized guidance as soon as possible to formulate a reasonable range of weight gain. By controlling diet, encouraging exercise, and paying more attention to the regulation of pregnant women’s endocrine and metabolic functions, the occurrence of GDM and perinatal complications can be prevented and controlled. In this retrospective study, single-center cohort data were used, the sample size for collection and analysis was small, and there were certain geographical limitations. It was unknown whether the pregnant women had undergone dietary intervention in the first trimester or before pregnancy. Relevant conclusions still need to be explored in a large-scale multicenter prospective cohort study. Under the circumstance of strictly controlling the interference factors, the feasibility of the results of this experiment can be further verified. Body composition standards should be formulated in line with various regions to guide clinical practice and further improve the quality of obstetric care for the birthing population.

## Data availability statement

The raw data supporting the conclusions of this article will be made available by the authors, without undue reservation.

## Ethics statement

The study was approved by the Ethics Committee of the Third Affiliated Hospital of Zhengzhou University, Henan Province (2022-143-01). Written informed consent for participation was not required for this study in accordance with the national legislation and the institutional requirements.

## Author contributions

LXT and CRM: data acquisition and drafting of the manuscript. LXT, ZL, HYD, CTT, GYY, and LJX: acquisition and analysis and interpretation of the data. XDM and CLL: study concept and design, critical revision of the manuscript for important intellectual content, and study supervision. All authors contributed to the article and approved the submitted version.

## Funding

This study was funded by the 2022 Henan Provincial Medical Science and Technology Research Program (LHGJ20220539) and the Key Research Project of Henan Higher Education Institution Scientific Research Projects (23A320066)

## Conflict of interest

The authors declare that the research was conducted in the absence of any commercial or financial relationships that could be construed as a potential conflict of interest.

## Publisher’s note

All claims expressed in this article are solely those of the authors and do not necessarily represent those of their affiliated organizations, or those of the publisher, the editors and the reviewers. Any product that may be evaluated in this article, or claim that may be made by its manufacturer, is not guaranteed or endorsed by the publisher.
